# PAI-1 and t-PA/PAI-1 complex potential markers of fibrinolytic bleeding after cardiac surgery employing cardiopulmonary bypass

**DOI:** 10.1186/1471-2253-12-27

**Published:** 2012-10-30

**Authors:** Agnese Ozolina, Eva Strike, Inta Jaunalksne, Angelika Krumina, Lars J Bjertnaes, Indulis Vanags

**Affiliations:** 1 Department of Anaesthesiology and Cardiac surgery, Pauls Stradins Clinical University Hospital, Pilsonu street 13, Riga, Latvia; 2 Department of Anaesthesiology and Reanimatology, Riga Stradins University, Dzirciema street 16, Riga, Latvia; 3 Clinical Immunology Centre, Pauls Stradins Clinical University Hospital, Pilsonu street 13, Riga, Latvia; 4 Department of Infectology and Dermatology, Riga Stradins University, Dzirciema street 16, Riga, Latvia; 5 Anaesthesia and Critical Care Research Group, Department of Clinical Medicine (Anaesthesiology), Faculty of Medicine, University of Tromsø, MH-Breivika, Tromsø, 9038, Norway; 6 Department of Anaesthesiology, University Hospital of North Norway, Sykehusveien 38, Tromsø, 9038, Norway

**Keywords:** Cardiac surgery, Fibrinolysis, Plasminogen activator inhibitor, Tissue plasminogen activator

## Abstract

**Background:**

Enhanced bleeding remains a serious problem after cardiac surgery, and fibrinolysis is often involved. We speculate that lower plasma concentrations of plasminogen activator inhibitor – 1 (PAI-1) preoperatively and tissue plasminogen activator/PAI-1 (t-PA/PAI-1) complex postoperatively might predispose for enhanced fibrinolysis and increased postoperative bleeding.

**Methods:**

Totally 88 adult patients (mean age 66 ± 10 years) scheduled for cardiac surgery, were enrolled into a prospective study. Blood samples were collected pre-operatively, on admission to the recovery and at 6 and 24 hours postoperatively. Patients with a surgical bleeding that was diagnosed during reoperation were discarded from the study. The patients were allocated to two groups depending on the 24-hour postoperative chest tube drainage (CTD): Group I > 500ml, Group II ≤ 500ml. Associations between CTD, PAI-1, t-PA/PAI-1 complex and D-dimer were analyzed with SPSS.

**Results:**

Nine patients were excluded because of surgical bleeding. Of the 79 remaining patients, 38 were allocated to Group I and 41 to Group II. The CTD volumes correlated with the preoperative plasma levels of PAI-1 (r = − 0.3, P = 0.009). Plasma concentrations of preoperative PAI-1 and postoperative t-PA/PAI-1 complex differed significantly between the groups (P < 0.001 and P = 0.012, respectively). Group I displayed significantly lower plasma concentrations of fibrinogen and higher levels of D-dimer from immediately after the operation and throughout the first 24 hours postoperatively.

**Conclusions:**

Lower plasma concentrations of PAI-1 preoperatively and t-PA/PAI-1 complex postoperatively leads to higher plasma levels of D-dimer in association with more postoperative bleeding after cardiac surgery.

## Background

Increased per – and postoperative bleeding remains to be a serious problem in cardiac surgery. Alterations in hemostasis per - and postoperatively may have a diversity of etiologies. These include the surgery per se as well as effects of the cardiopulmonary bypass (CPB) on the coagulation and the inflammation cascades, and their cross-reactions with the fibrinolytic – and the kinin-kallikrein systems [1-3]. During the last few years, increasing attention has been paid to reports demonstrating the influence of the fibrinolytic system on increased bleeding, particularly after cardiac surgery employing CPB [1,4-6].

Plasminogen, alpha-2 antiplasmin, tissue plasminogen activator (t-PA) and urinary type plasminogen activator are the main fibrinolytic components of plasma. The generation of plasmin is mainly regulated by processes involving t-PA and its counterpart plasminogen activator inhibitor type – 1 (PAI-1), which blocks the conversion of plasminogen to plasmin, thus inhibiting fibrinolysis [7,8]. PAI-1 is a serine protease, which is synthesized in platelets as well as in endothelium and adipose tissues [9]. PAI-1 binds rapidly with a ratio of 1:1 to t-PA forming a stable t-PA/PAI-1 complex, which is cleared from the circulation by macrophages in the liver. The rate of formation of the t-PA/PAI-1 complex depends on the plasma concentrations of the two proteins: the higher the concentrations of t-PA and PAI-1, the more complex will be formed in the circulation [10].

Cardiac surgery employing CPB is associated with increased fibrinolytic activity and enhanced concentrations of PAI-1 and D-dimer as compared to off-pump surgery [11-13]. However, inter-individual variations in PAI-1 and t-PA/PAI-1 complex formation are relatively large. After normal primary hemostasis, low PAI-1 and low t-PA/PAI-1 complex plasma concentrations, may result in hyperfibrinolytic hemorrhage [8]. This implies that clots are primarily formed, but fibrinolysis occurs readily since the half-life of PAI-1 is short and the process might lead to relative lack of inhibitor to abate the plasmin activity.

We hypothesize that control of the fibrinolytic system pre – and postoperatively strengthen the possibilities of predicting enhanced bleeding after cardiac surgery. Therefore, our aim was to assess fibrinolytic activity pre- and postoperatively in patients undergoing cardiac surgery with the use of CPB.

## Methods

The study protocol and the informed consent form were approved by the Ethics Committee (No.151209-4L) of Pauls Stradins Clinical University Hospital, Riga, Latvia. Written informed consent was obtained from every patient.

### Population

Between 1 May and 30 December 2010, 88 consecutive adult patients, who were admitted to the hospital to undergo cardiac surgery by the use of CPB, were considered for a prospective observational study. None of the patients received antifibrinolytic medicines during - or after the surgery.

### Inclusion and exclusion criteria

*Inclusion criteria*: > 18 years of age, first-time coronary artery bypass grafting (CABG) and/or valve replacement under CPB, EuroSCORE [14] < 10%, coagulation tests within normal ranges at baseline {prothrombin time (PT) 70-120%} or international normalized ratio (INR) 0.8-1.2, fibrinogen plasma concentration 1.5 – 3.5 g/L, platelet count (PLT) 150 – 400 × 10^9^/L, hemoglobin (Hb) concentration > 135 g/L for men and > 120 g/L for women) and no anticoagulant, - anti-aggregating or non-steroidal anti-inflammatory drugs for, at least, five days prior to surgery in order to disclose drug-induced platelet dysfunction. The last dose of low-molecular-weight heparin (LMWH) was administered the evening before the surgery. *Exclusion criteria*: emergency - and redo operations, preoperative hemostatic disorders with a history of hemorrhagic events or coagulopathy (PT below 50% or INR greater than 1.5, fibrinogen plasma concentration below 1.5 g/L, PLT lower than 100 × 10^9^/L) and severe renal and/or hepatic dysfunctions.

### Perioperative management

Anesthesia was induced with fentanyl (Fentanyl-Kalceks® 0.05 mg/ml, A/S Kalceks, Latvia), 0.2-0.3 mg, midazolam (Dormicum®, F. Hoffman-La Roche AG, Switzerland), 2.5-5 mg, propofol (Propofol-Lipuro® 10mg/ml, B. Braun Melsungen AG, Germany) 1–3 mg/kg and cisatracurium (Nimbex® 2 mg/ml, GlaxoSmithKline Manufacturig S.p.A, Italy) 0.2 mg/kg intravenously and maintained with inhalation of sevoflurane (Sevoflurane Piramal®, Piramal Healthcare Ltd, United Kingdom) at 0.8-1.2 MAC. Before the start of CPB, heparin (Pan-Heparin Sodium®, Panpharma S.A./Rotexmedica Gmbh, Germany) was administered at a dose of 300–400 units/kg followed by 5.000 - 10.000 units to maintain an activated coagulation time (ACT) above 480 seconds. During CPB (Admiral®, Eurosets TM, Italy), anesthesia was maintained with fentanyl 0.03-0.06 mkg/kg/min, propofol 3–5 mg/kg/h and cisatracurium 0.1 mg/kg/h. Patients were cooled to a bladder temperature of 34–35°C. Myocardial protection was achieved by using St. Thomas 4:1 cardioplegia (AlleMan®, Germany). Weaning off CPB after the surgery was performed after the patient was rewarmed to a bladder temperature above 36°C. After separation from CPB, protamine (Protamin Meda®, Meda Pharma, Austria) was administered at a dose of 1 mg per 100 units of heparin followed by additional doses until ACT had returned to baseline. Postoperatively, standard unfractionated heparin was administered from 20–24 hours after valve surgery. Warfarin (Orfarin®, Orion Pharma, Finland) treatment was resumed on the third postoperative day if the patient had no signs of bleeding or need for re-operation. LMWH (Fragmin® 2500 IU/1ml, Pfizer, Belgium) was also started 20–24 hours after CABG in patients without increased bleeding tendency. According to our clinical guidelines, a hematocrit < 26% indicated requirement for transfusion of packed erythrocytes; PT < 50%, and PLT < 90 × 10^9^/l indicated need for transfusion of freshly frozen plasma and platelet concentrates, respectively. In conditions of increased bleeding with drop in fibrinogen, cryoprecipitate was given.

### Demographic and laboratory data

We noticed the following demographic and perioperative variables: age, sex, body mass index (BMI), ejection fraction (EF), comorbidities, preoperative anticoagulation therapy, type of surgery, extracorporeal circulation time (min), aortic clamp - and reperfusion times, and transfusion requirements. Moreover, we analyzed PAI-1 preoperatively and t-PA/PAI-1 complex 24 hours postoperatively. PAI-1 (normal range 1–25 ng/ml) and t-PA/PAI-1 complex (normally < 5 ng/ml) were determined by using enzyme-linked immunosorbent assay (ZYMUTEST, HYPHEN BioMed, France). Cross-linked fibrin degradation products (D-dimer, normally < 300 ng/ml) were quantified with the immunoturbidimetric test (D-dimer PLUS, Dade Behring, Marburg, Germany). Fibrinogen plasma concentration was determined according to Clauss [15]. PT was analyzed with a prothrombin complex assay (Lyophilized Dade® and Innovin®, Siemens Healthcare Diagnostics, USA). All the coagulation tests were determined using Sysmex® CA-1500 (Siemens Healthcare Diagnostics, Germany). Hb and PLT were analyzed by means of a Beckman Coulter LH 750 Hematology Analyzer.

### Groups of patients

Bleeding volume was recorded as milliliters of chest tube drainage (CTD) 24 hours (h) postoperatively and the patients were allocated to two groups; Group I: bleeding arbitrarily defined as CTD > 500 ml/24h and Group II with CTD ≤ 500 ml/24h. Indication for reoperation because of suspected surgical bleeding was based on evaluation of clinical and hemodynamic changes. If the patient was re-operated, the CTD volume until reoperation, and 24 hours afterwards was registered. A surgical bleeding was diagnosed only if one or more specific bleeding sites were identified. Then, the patient was excluded from further study. If no specific site was located, the bleeding was registered as hemostatic disorder and the patient was allocated to the most appropriate group according to the bleeding volume. Plasma fibrinogen, PT, PLT and Hb were assessed preoperatively (T0) and together with D-dimer upon admission to the intensive care unit (T1), and at 6 and 24 hours (T6, T24) postoperatively.

### Statistical analysis

Data was analyzed with SPSS (SPSS® version 17.0, Chicago, IL). Continuous variables were presented as mean ± standard deviation (SD) and categorical variables as percentages (%). Linear regression (Pearson^’^s correlation coefficient) was used to analyze the relationships between the demographic and the surgical data and the hematologic -, coagulation – and fibrinolysis parameters (PAI-1, t-PA/PAI-1 complex and D-dimer) and bleeding volumes, respectively. Comparisons between the groups were performed with Mann–Whitney U test for non-parametric variables, and two-sample t test or ANOVA for parametric variables. Chi-square test was used to analyze categorical data. Statistical significance was defined as a P< 0.05.

## Results

### Clinical course

Totally 88 consecutive patients (47 men and 41 women) 66 ± 10 years (mean ± SD) of age scheduled for cardiac surgery were considered for inclusion. Eleven patients (12.5%) required reoperation between 10 minutes and 62 hours postoperatively, seven of them within 24 hours because of suspected surgical bleeding or hemipericardium, and they all survived. A surgical bleeding site was identified in nine patients (10.2%) that were discarded from further data analysis. In two patients, a specific site was not found and they were allocated to Group I. As surveyed in Table 1, 79 patients were subjected to further analysis: 38 patients were diagnosed with a CTD > 500 ml/24h (Group I), whereas 41 patients were registered with a postoperative CTD ≤ 500 ml/24h (Group II). We found significant differences in 24 hour CTD and transfusion requirements between the groups. Moreover, we noticed no significant intergroup differences in demographic characteristics (mean age, gender, BMI, EF, comorbidities, preoperative medication or surgical variables) or in preoperative coagulation tests (PT, APTT, fibrinogen and PLT).

**Table 1:** **Demographic characteristics and co**-**morbidities of patients with postoperative bleeding after cardiac surgery employing cardiopulmonary bypass**

**Variables**	**I Group n=38**	**II Group n=41**	**P value**
**Demographic**			
Sex (male/female)	21/17	17/24	0.5
Age (years)	67 ± 10	66 ± 10	0.7
EuroSCORE (%)	4.8 ± 2	4.9 ± 1.7	0.2
BMI (kg/m2)	27.6 ± 4.8	28.3 ± 4.6	0.5
EF (%)	55 ± 8.8	56 ± 6.7	0.2
Hypertension (n)	13	22	0.1
Previous myocardial infarction (n)	13	13	1.0
Hypercholesterolemia (n)	6	11	0.2
Pulmonary obstructive disease (n)	5	3	0.48
**Preoperative medication**			
Aspirin (n)	26	29	0.7
Clopidogrel (n)	6	9	0.44
LMWH (n)	31	25	0.42
Warfarin (n)	2	1	0.6
**Preoperative coagulation tests**			
PT (%)	87 ± 15	92 ± 12	0.15
APTT (sec.)	34 ± 6	32 ± 4	0.05
Fibrinogen (g/L)	4.4 ± 1.1	4.7 ± 1.5	0.25
PLT (x10^9^/L)	205 ± 39	225 ± 72	0.24
**Surgical parameters**			
CABG (n)	16	17	0.9
Valve replacement (n)	13	16	0.6
Combined surgery (n)	8	9	0.8
CPB duration (min)	106 ± 40	102 ± 41	0.8
Aorta occlusion time (min)	66 ± 25	64 ± 29	0.4
Reperfusion time (min)	35 ± 15	32 ± 15	0.5
Blood loss 24h (ml)	812 ± 269	346 ± 102	<0.001
Erythrocyte requirements during ICU stay (n)	14	3	0.008
Plasma requirements during ICU stay (n)	14	1	0.001
Cryoprecipitate requirements during ICU stay (n)	7	1	0.03

Data are presented as mean  ±  SD or number (n).

BMI, body mass index; EF, ejection fraction; LMWH, low molecular weight heparin; PT, prothrombin time; APTT, activated partial thromboplastin time; PLT, platelet count; CABG, coronary artery bypass grafting; CPB, cardiopulmonary bypass; ICU, intensive care unite; n, number of patients.

### Variables of fibrinolysis

**Figure 1:**
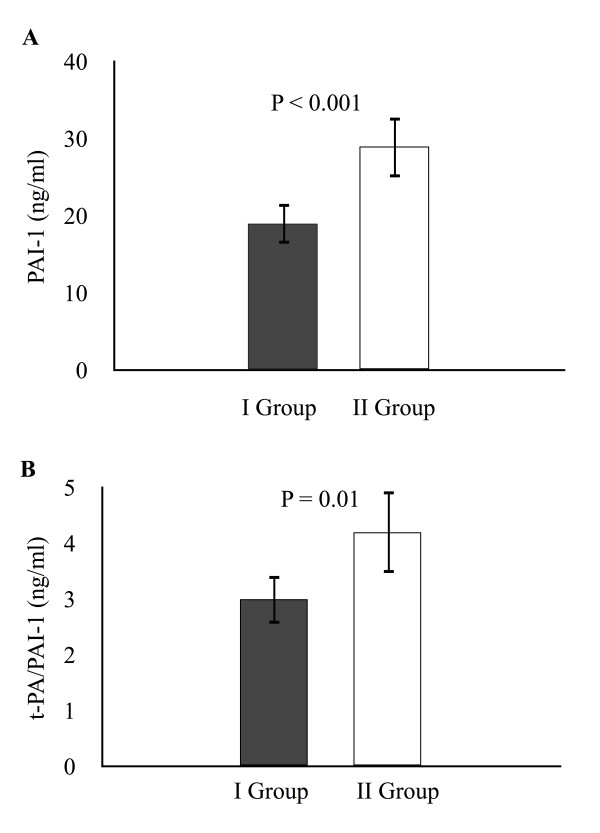
**(A)Preoperative plasma concentration of plasminogen activator inhibitor type-1.** (**B**) Postoperative plasma concentration of tissue plasminogen activator/plasminogen activator inhibitor type-1 complex. Group I, patients with postoperative blood loss of > 500 ml/24 hours; Group II, patients with postoperative blood loss of ≤ 500 ml/24 hours PAI-1, plasminogen activator inhibitor type − 1; t-PA-PAI-1: tissue, plasminogen activator/plasminogen activator inhibitor type-1 complex. Data are presented as mean ± SD.

**Figure 2:**
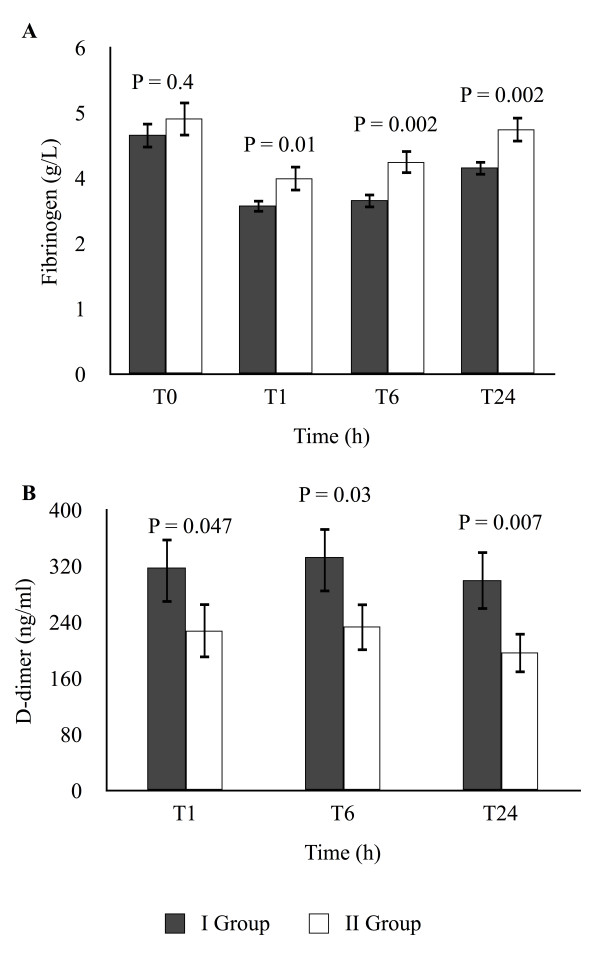
**(A)Plasma concentrations of fibrinogen: at baseline (TO), upon admision to the intensive care unit (T1), six hours after surgery (T6) and twenty-four hours after surgery (T24).** (**B**) Postoperative plasma concentrations of D-dimer: upon admission to the intensive care unit (T1) six hours after surgery (T6) and twenty-four hours after surgery (T24). Group I, patients with postoperative blood loss of >500 ml/24 hours; Group II, patients with postoperative blood loss of ≤ 500 ml/24 hours. Data presented as mean ± SD.

As shown in Figure 1, both the mean plasma concentration of PAI-1 preoperatively (A) and of t-PA/PAI-1 complex 24 hours postoperatively (B), were lower in Group I (P < 0.001 and P = 0.01, respectively). Postoperatively, mean plasma concentrations of fibrinogen increased less from T1 and beyond in Group I as compared to Group II (P = 0.01) as depicted in Figure 2A. Concomitantly, the mean plasma concentrations of D-dimer were significantly higher (P < 0.05) in Group I as compared to Group II (Figure 2B).

### Associations between postoperative bleeding and variables of fibrinolysis

**Figure 3:**
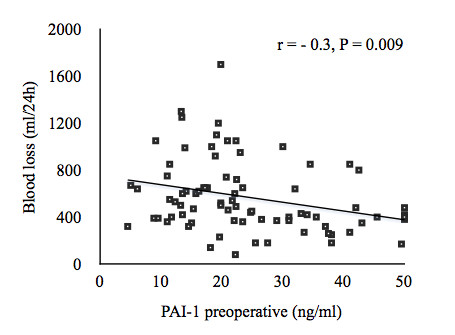
**Relationships between preoperative plasma concentrations of plasminogen activator inhibitor type** - **1 and blood loss over 24 hours of patients subjected to cardiac surgery employing cardiopulmonary bypass.** *P < 0.05. PAI-1: plasminogen activator inhibitor type - 1.

We found no correlation between demographic and surgical parameters and the plasma concentrations of PAI-1 and t-PA/PAI-1 complex. However, preoperative PAI-1 levels correlated inversely with 24 hour postoperative blood loss (Figure 3; r = − 0.3, P = 0.009). In contrast, the correlation between t-PA/PAI-1 complex and 24 hour blood loss did not reach statistical significance (r = − 0.24, P = 0.08).

## Discussion

The present study revealed that lower levels of PAI-1 preoperatively and of t-PA/PAI-1 complex postoperatively, are associated with lower plasma concentrations of fibrinogen, higher levels of D-dimer and increased blood loss during the first 24 hours after the operation. Our results are consistent with several recent investigations showing that activation of the fibrinolytic system is associated with increased postoperative bleeding in cardiac surgery employing CPB [1,5,12].

The plasma concentrations of PAI-1 and t-PA/PAI-1 complex that are supposed to be the main regulators of fibrinolysis in humans, are both characterized by wide variations that may explain the large inter - individual differences in fibrinolytic activity [16]. Several recent studies have described the influence of genetic factors, such as PAI-1 promoter – 675 (4G/5G) polymorphism, on the plasma levels of PAI-1, t-PA and t-PA/PAI-1 complex [1,4,6,17-20]. The 5G allele is associated with low levels of PAI-1 [4,6,17,19].

Our patients were cooled on CPB to a bladder temperature of 34–35°C with no intergroup difference. Some investigators claim that reduced temperature lowers endogenous production of PAI-1, resulting in enhanced fibrinolysis and increased per- and postoperative bleeding [21] whereas others refute this idea [22]. Our patients were rewarmed to normal body temperature before transfer to the recovery. Therefore, it is unlikely that temperature had any influence on the formation of t-PA/PAI-1 complex. Since PAI-1 is a more stable indicator of fibrinolysis, as compared to t-PA, whose concentration peaks during CPB, we determined PAI-1 before the operation and t-PA/PAI-1 complex after the surgery [23] as well as their associations with postoperative blood loss 24 hours after the surgery. Our results indicated that those presenting with higher preoperative plasma concentrations of PAI-1 had less blood loss, and conversely, those with a lower preoperative plasma level had a larger blood loss 24 hours after surgery. Other investigators have noticed similar results [1,24]. Recently, investigators have reported favorable effects of administration of very long half-life PAI-1 (> 700 hours) on bleeding time and total blood loss after tail clip in PAI-1 deficient mice [25]. However, so far, very long half-life PAI-1 is not available as a medicine to promote hemostasis after surgery, trauma, or PAI-1 deficiency in humans.

As to the best of our knowledge, the literature is scanty on reports focusing on the importance of t-PA/PAI-1 complex and its relationship with enhanced bleeding after CPB. Our notion that patients with an accumulated blood loss in excess of 500 ml 24 hours after CPB had lower levels of t-PA/PAI-1 complex is consistent with the findings reported by Rivera and coworkers [1]. In a subgroup of patients presenting with enhanced bleeding, these workers reported lower levels of PAI-1 both before - and after surgery and lower concentrations of t-PA/PAI-1 complex postoperatively. Surprisingly, we observed no significant correlation between the preoperative concentration of PAI-1 and the level of t-PA/PAI-1 complex 24 hours postoperatively. We speculate that the lack of such correlation can be explained by the fact that the increase in PAI-1 production culminates on the first postoperative day and usually returns towards normal on the second postoperative day [23]. The plasma levels of PAI-1 are known to increase immediately after CPB as part of the „fibrinolytic shut-down” [26] and afterwards, it decreases slowly over the subsequent days or weeks [27].

Lower preoperative levels of PAI-1 and lower t-PA/PAI-1 complex ratio 24 hours after surgery might have led to higher levels of D-dimer immediately after the surgery [28,29]. Kuepper and co-workers [5] examined 120 patients scheduled for cardiac surgery who were randomized to an aprotinin group and a control group. D-dimers reached higher plasma levels in the control group indicating increased fibrinolysis. Consistently, in our study, the patients of Group I (with the highest blood loss) had significantly lower fibrinogen - and higher D-dimer levels after surgery and throughout the ensuing 24 hours (Figure 2). The significantly lower fibrinogen level in Group I immediately after the surgery might indicate increased consumption because of the hyperfibrinolytic state of this group. These findings are also supported by the contention of previous workers that plasmin generation and fibrin degradation is increased 10-to 20-fold during CPB, and moreover that fibrin formation and degradation rates are nearly equally affected by the CPB [2,30].

Our study has limitations. That we found no associations between preoperative PAI-1 and postoperative t-PA/PAI-1 complex could be due to a small sample size. By considering the first 25 patients included in each group, calculation of sample size revealed that the correlation between t-PA/PAI-1 complex and blood loss after 24 hours (r = − 0.24, P = 0.08) was underpowered and might have reached statistical significance (P<0.05 and a power of 80%) first by increasing the total number of patients to 134 (n = 67 in each group). Another weakness is that we did not analyze patient outcome data.

An attractive idea for a future investigation would be to determine t-PA and PAI-1 activity separately and in concert with t-PA/PAI-1 complex concentrations. This would increase our understanding of the relationship between free PAI-1 antigen and its formation of t-PA/PAI-1 complex. Moreover, a multicenter study should be performed focusing on the influence of the fibrinolytic system on postoperative bleeding and its relation to outcome after cardiac surgery.

## Conclusions

Taking into account the complexity of enhanced bleeding after cardiac surgery, it might be difficult to isolate one factor as the denominator of bleeding. Our investigation indicates that low plasma levels of PAI-1 preoperatively and of t-PA/PAI- 1 complex postoperatively, in parallel with increased plasma concentration of D-dimer can be useful predictors of fibrinolysis, and thus, of increased postoperative blood loss. Therefore, by including screening of fibrinolytic markers pre – and postoperatively, we might be able to identify patients with low fibrinolytic inhibitory potential who might benefit from antifibrinolytic therapy prior to cardiac surgery.

## Abbreviations

ACT: Activated coagulation time; BMI: Body mass index; CABG: Coronary artery bypass grafting; CPB: Cardiopulmonary bypass; CTD: Chest tube drainage; EF: Ejection fraction; EuroSCORE: European System for Cardiac Operative Risk Evaluation; Hb: Blood hemoglobin concentration; ICU: Intensive care unit; INR: International normalized ratio; LMWH: Low-molecular-weight heparin; PAI-1: Plasminogen activator inhibitor type-1; PLT: Platelet count; PT: Prothrombin time; t-PA: Tissue plasminogen activator; t-PA/PAI-1: Tissue plasminogen activator/plasminogen activator inhibitor type-1 complex; SD: Standard deviation; T0: Preoperatively; T1: On admission to the intensive care unite; T6: Six hours after surgery; T24: Twenty-four hours after operation; u-PA: Urinary type plasminogen activator.

## Competing interests

The authors declare that they have no competing interests.

## Authors’ contributions

AO and ES conceived the study. ES was responsible for anesthesia and applied the same anesthetic procedure in all the patients. AO participated in the design and the administration of the study, informed the patients and obtained their written consent. IV, AK and AO collected the clinical and laboratory data for analysis. IJ carried out the coagulation and fibrinolysis immunoassays and interpreted the results. AO and ES performed the statistical analysis and interpreted the data. AO, IV, ES and LJB drafted the manuscript. All authors read and approved the final manuscript.

## Pre-publication history

The pre-publication history for this paper can be accessed here:

http://www.biomedcentral.com/1471-2253/12/27/prepub
